# Dermal cutaneous clear cell sarcoma defined by novel EWSR1-CREM fusion

**DOI:** 10.1016/j.jdcr.2024.06.026

**Published:** 2024-07-23

**Authors:** Cheyenne J. Hornback, Jessica Cintolo-Gonzalez, Philip E. LeBoit, Laura A. Greene, Julia Barker

**Affiliations:** a Division of Dermatology, Dermatology Resident from the University of Vermont Medical Center, Burlington, Vermont; b Division of Surgical Oncology, Assistant Professor of Surgical Oncology from the University of Vermont Medical Center, Burlington, Vermont; c Department of Pathology and Dermatology, Professor of Pathology and Dermatology Division Chief of UCSF Dermatopathology and Oral Pathology Service from the University of California San Francisco, San Francisco, California; d Department of Pathology and Laboratory Medicine, Professor of Pathology and Director Pathology Residency Program from the University of Vermont Medical Center, Burlington, Vermont; e Division of Dermatology, Assistant Professor of Dermatology from the University of Vermont Medical Center, Burlington, Vermont

**Keywords:** clear cell sarcoma, EWSR1-CREM, melanoma of the soft parts

## Introduction

Clear cell sarcoma (CCS) is a rare neoplasm of mesenchymal derivation originating in deep tissues associated with tendons or aponeuroses of the distal extremities.^1^, ^2^, ^3^ CCS was first described by Enzinger in 1965 as having morphologic and histopathologic overlap with malignant melanoma and it was colloquialized as malignant melanoma of the soft parts.^1^, ^2^, ^3^, ^4^ Cutaneous CCS is a rare entity with localization to the dermis and subcutis.^5^

The histopathologic overlap between melanoma and cutaneous CCS presents a diagnostic challenge for pathologists. Molecular genetic techniques have afforded indisputable evidence that CCS is genetically distinct from malignant melanoma due to the cytogenic hallmarks of t(12;22) (q13;q12) or less commonly a t(2;22) (q34;q12) translocation, forming chimeric *EWSR1::ATF1* or *EWSR1::CREB1* fusions in CCS, which are not found in melanoma.^2^

Here, we present a diagnostic challenge in which a cutaneous CCS was misdiagnosed as acral melanoma. With the assistance of next-generation sequencing, a novel case of cutaneous dermal-based clear cell sarcoma with *EWSR1::CREM* fusion was discovered.

## Case

**Fig 1:**
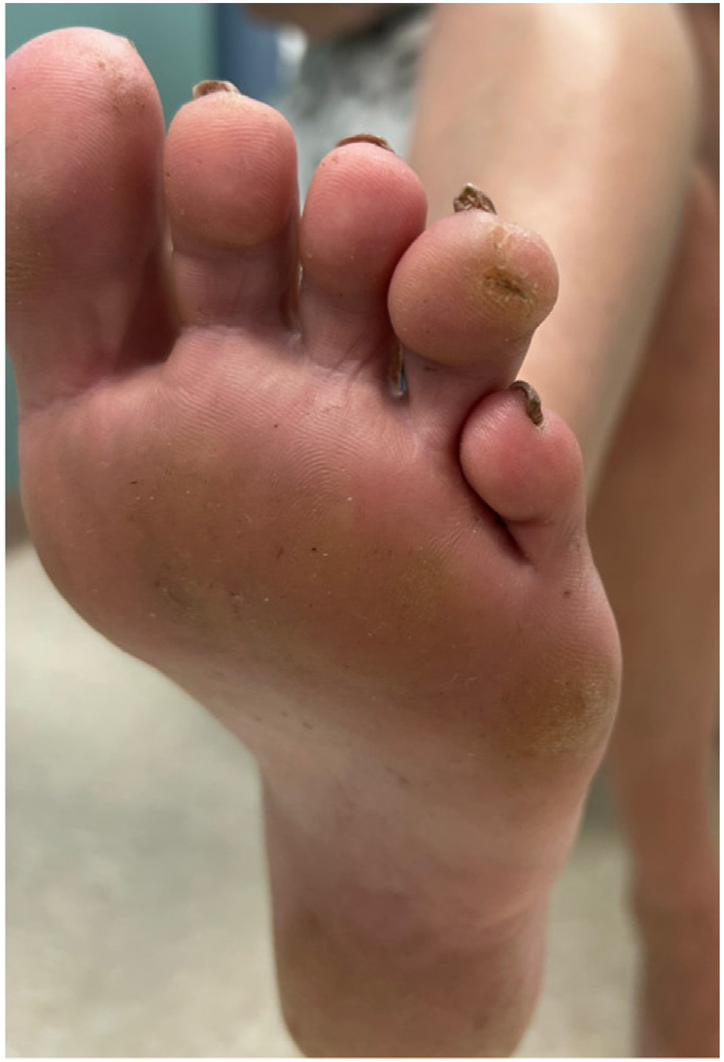
Preoperative photo of the bulbous indurated subcutaneous nodule of the left lateral fourth toe

**Fig 2:**
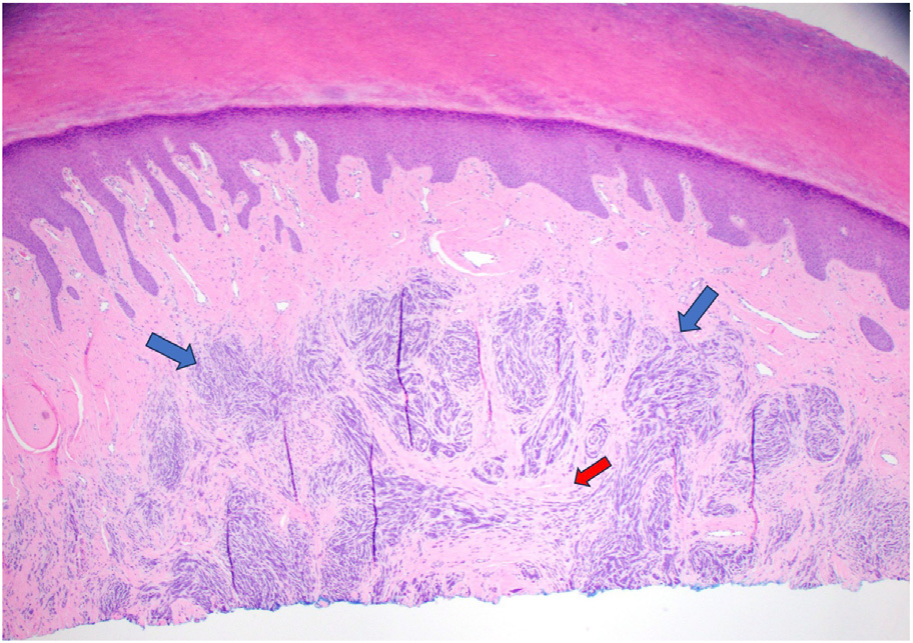
H&E 4×, shave biopsy specimen with hyperchromatic basaloid cells (*blue arrows*) arranged as nests, strands, and cords within the dermis in a swirled pattern with myxoid stroma (*red arrow*).

**Fig 3:**
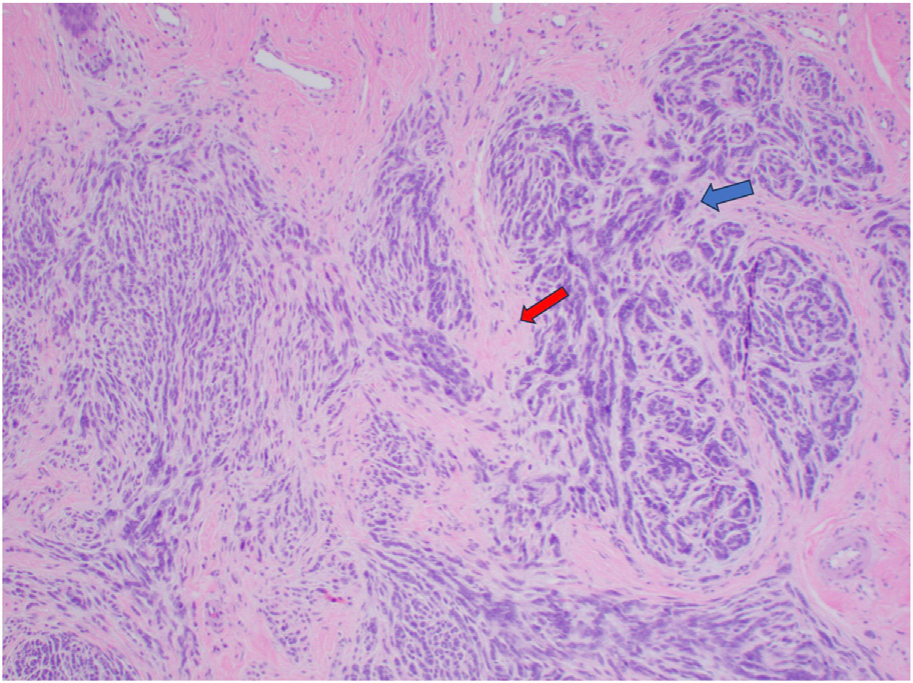
H&E 10×, shave biopsy specimen with nests of basaloid cells (*blue arrow*) with pleomorphism, prominent nucleoli, and myxoid stroma (*red arrow*).

A 67-year-old woman presented with an asymptomatic callus for at least 12 years. Examination revealed a bulbous indurated subcutaneous nodule on the left fourth toe (Fig 1). Prior to biopsy, radiographs ruled out intraosseous involvement. Histopathologic examination revealed hyperchromatic basaloid cells arranged as nests, strands, and cords within the dermis. The cells had a swirled pattern with myxoid stroma. The epidermis was not involved. SOX-10 was diffusely positive (Fig 2). MART-1 and HMB45 showed focal strong positivity, but the majority of the basaloid cells were negative. The inconclusive findings were sent for expert opinion. Expert one interpreted the lesion as a neurotized intradermal nevus with perineural features. It showed immunoreactivity for S-100 protein but negative for PRAME. The case was then sent for a second consultation with molecular testing. FISH showed an 11q gain and was diagnosed as a melanoma of at least 2.1 mm in thickness (Fig 3).

**Fig 4:**
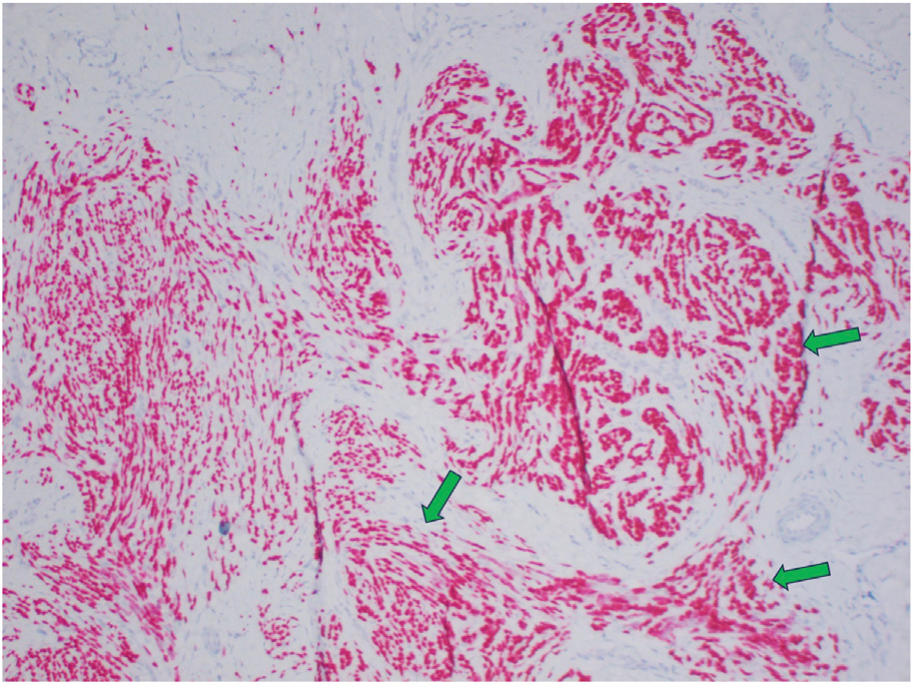
SOX-10 staining is positive (*green arrow*) in the basaloid dermal proliferation.

However, due to the lack of morphologic features to support the diagnosis of melanoma and unusual clinical history, next-generation sequencing was performed (UCSF500 panel, Clinical Cancer Genomics Laboratory, University of California, San Francisco), which showed an *ESWR1::CREM* gene fusion, favoring CCS rather than desmoplastic melanoma (Fig 4). Given the cytologic features, immunohistochemical staining pattern, and genetic testing, we favored CCS.

Due to the potential for aggressive behavior, a preoperative positron emission tomography-computed tomography scan demonstrated focal uptake of left fourth toe with no evidence of metastasis. Sentinel lymph node biopsy was negative. The toe was amputated to obtain a 2 cm tissue margin. Histologic examination showed residual tumor approaching periosteum with focal perineural invasion with similar histologic and immunohistochemical findings to initial biopsy, interpreted as residual CCS with *EWSR1::CREM* gene fusion. While most CCSs are aggressive, this case demonstrated indolence. The patient will be followed closely to assess for recurrence or metastatic disease. Intradisciplinary tumor board decided that no adjuvant therapy is needed.

This was a challenging case sent in consultation to 2 institutions with differing opinions. Without molecular testing, a diagnosis of CCS would have been unattainable.

## Discussion

CCS is a rare, aggressive tumor that accounts for 1% of all sarcomas.^5^, ^6^, ^7^ Cutaneous CCS usually evolves from large deep-seated lesions, but a rarer superficial subtype originating from the dermis without intraepidermal involvement has been described.^3^^,^^5^ Diagnosis of cutaneous CCS is often delayed due to indolent behavior.^5^ The differential diagnosis includes cellular blue nevi, perivascular epithelioid cell neoplasm, melanocytic tumors with *CRTC1::TRIM11* fusions, melanoma, and epithelioid malignant peripheral nerve sheath tumor.^5^^,^^8^

Cutaneous melanocytic tumors with *CRTC1:TRIM11* fusion are a rare entity that can overlap with CCS. Cutaneous melanocytic tumors with *CRTC1:TRIM11* fusion typically presents as a slow-growing, asymptomatic intradermal, or subcutaneous nodule. They were historically thought to be a CCS with a novel fusion, but are now known as a separate entity.^8^ Overlapping pathologic features include nests and fascicles of epithelioid or spindled tumor cells, pale to eosinophilic cytoplasm, and focal invasion into the subcutis with possible involvement of the epidermis. Distinction between the two is crucial, as Cutaneous melanocytic tumors with *CRTC1:TRIM11* fusion is typically a low-grade neoplasm with good prognosis. However, a case with distant pulmonary metastasis was reported.^9^ CCS portends high recurrence rates, metastasis, and tumor-related death.^8^

Although a tumor of sarcomatous origin, the most difficulty lies within the distinction of CCS from melanoma.^6^ While the absence of a junctional component has been used traditionally to distinguish CCS from melanoma, we now know that this feature can occur in occasional cases of CCS.^6^ Like melanoma, CCS cells can contain melanin pigment.^4^ CCSs can have multinucleated wreath-like giant cells, but these can also be seen in melanoma.^4^

Due to the histologic overlap with malignant melanoma, most CCSs are diagnosed through molecular testing. Next-generation sequencing was prompted because the histologic findings were not diagnostic of any one entity but rather demonstrated characteristics of several diagnoses on the differential. The histologic findings that prompted further testing include nests of basaloid cells with melanocytic differentiation and a swirled myxoid stroma and convoluted immunohistochemical staining. The lesion stained diffusely positive with SOX-10 but was only focally positive for MART-1 and HMB-45 with the majority of the basaloid cells staining negative. It stained entirely negative for PRAME, which is unusual for a diagnosis of melanoma. We recommend considering next-generation sequencing if a definitive diagnosis cannot be elucidated, especially if not correlating clinically. With next-generation sequencing, the majority of CCSs exhibit the characteristic reciprocal translocation t (12;22) involving *EWSR1::ATF1* while a small subset is identified by *EWSR::CREB1*.^3^ Only two other cases containing the *EWSR::CREM* fusion have ever been identified.^3^ None of the reported cases consist of dermal-based CCS.^3^^,^^4^^,^^6^

CCS is usually an aggressive tumor that is associated with local recurrence and late metastases.^2^^,^^4^^,^^6^ However, our case demonstrated an indolent course. Another case of *EWSR1::CREM*-positive cutaneous CCS was also associated with slow growth and delayed diagnosis with postsurgery recurrence within a regional lymph node and subsequent lung metastasis.^10^ The presence of *the EWSR1::CREM* fusion may be suggestive of a slow, progressive course, with late-stage metastasis.^10^

Wide local excision is the standard of care for cutaneous CCSs. Chemotherapy and radiotherapy have not been proven to be beneficial for patients, especially after disease has metastasized.^7^ With the overlap between CCS and melanoma, the success of immunotherapy in melanoma has prompted trials to assess whether the same clinical benefit can be observed in CCS. Thus far, pembrolizumab has produced durable responses in a small number of patients. Further investigation is needed, and clinical trials are ongoing to evaluate the efficacy of these agents in treating CCS.^11^

This case highlights the diagnostic challenges of cutaneous CCS, the histologic overlap with melanoma, and the necessity of molecular studies for proper diagnosis. The *ESWR1::CREM* fusion has never been previously identified in dermal-based CCS. Because of the rarity of CCS, further characterization of these tumors is necessary for accurate and early diagnoses, leading to improved patient outcomes.

## Conflicts of interest

None disclosed
